# Sonidegib after vismodegib discontinuation in a patient with Gorlin–Goltz syndrome and multiple basal cell carcinomas

**DOI:** 10.1111/dth.15095

**Published:** 2021-08-19

**Authors:** Alfredo Piccerillo, Alessandro Di Stefani, Alessio Costantini, Ketty Peris

**Affiliations:** ^1^ Institute of Dermatology Università Cattolica del Sacro Cuore Rome Italy; ^2^ Fondazione Policlinico Universitario A. Gemelli—IRCCS Rome Italy

Dear Editor

Nevoid basal cell carcinoma syndrome (NBCCS), also referred as Gorlin–Goltz syndrome, is a rare, autosomal‐dominant syndrome, caused by abnormalities in the sonic hedgehog signaling pathway and characterized by a multitude of basal cell carcinomas (BCCs), usually developed in a young age.^1^

Treatment of BCCs in patients with NBCCS can be extremely challenging due to the high burden of tumors that require multiple surgical excisions, often resulting in disfigurement and emotional distress.^2^ Radiotherapy use in NBCCS patients is controversial because of the carcinogenic effect of x‐rays that could lead to the formation of new BCCs.^3^ In patients who are not candidate for surgery or other treatment approaches, systemic therapy with hedgehog pathway inhibitor (HPI) is recommended, even if reimbursed only in case of advanced and/or metastatic tumors.^4^ Vismodegib and sonidegib are specific inhibitors of smoothened, a crucial oncogenic protein of the hedgehog pathway, currently approved for the treatment of advanced BCC; both drugs have been investigated in patients with NBCCS, showing objective response rate in about 60% of patients, even if associated with poor tolerability.

We report the case of an 89‐year‐old woman with NBCCS examined for the presence of a large ulcerated BCC located on the chin, causing continues bleeding and quality of life impairment. NBCCS diagnosis was established by the presence of three major criteria (multiple BCCs, odontogenic keratocysts, and family history of NBCC) and one minor criteria (Patched mutation).

Patient's medical history showed that she previously underwent multiple surgical excisions and had been successfully treated with vismodegib 150 mg/die for one large ulcerated BCC on the right nasal fold, one ulcerated BCC on the upper lip and an infiltrating BCC on the chin. The patient also had >50 superficial BCCs on the vertebral region. After 6 months of vismodegib, severe asthenia led to drug discontinuation.

The patient was lost to follow‐up and was visited again 36 months after the vismodegib interruption because of relapse of all BCCs previously treated, in particular one large ulcerated lesion located on the chin. Physical examination also showed two large ulcerated nodules located on the right nasal fold and upper lip (Figure 1A) which were considered noneligible to surgery and multiple superficial BCCs on the back (Figure 1B). Since according to the Italian rule we cannot prescribe again a HPI after a discontinuation lasting more than 2 months, treatment with sonidegib (200 mg/die) was initiated after approval of the Italian Medical Agency and patient's written informed consent.

**FIGURE 1:**
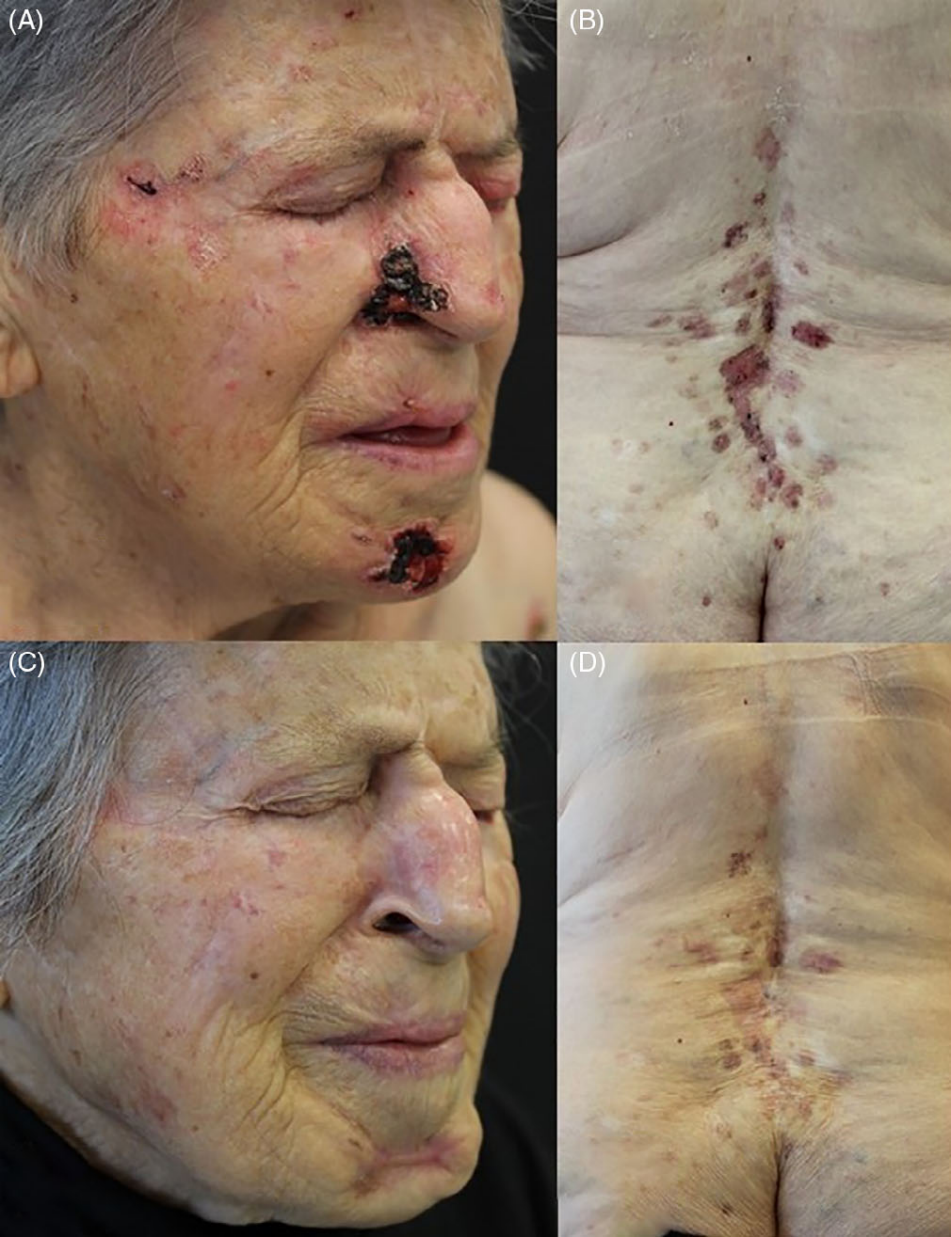
Multiple BCCs in a 89‐year old woman affected with NBCCS. (A) nodular, ulcerated BCCs on the right nasal fold, chin region and upper lip and (B) multiple superficial BCCs on the vertebral region. Efficacy after 6 months of treatment with sonidegib 200 mg/die. (C) complete healing of the BCCs on the face and (D) partial re‐epithelization of the multiple BCCs on the vertebral region. BCCs, basal cell carcinomas; NBCCS, nevoid basal cell carcinoma syndrome

After 3 months of sonidegib treatment, partial re‐epithelization and shrinkage in all the target lesions was observed, with no drug‐related adverse events (AE). Further improvement, with complete healing of the BCCs on the face (Figure 1C) and partial re‐epithelization of the BCCs on the back (Figure 1D) has been assessed after 6 months. To date, sonidegib treatment is continued with maintenance of clinical benefit and no drug‐related AE.

Management of BCCs in patients who experienced tumor recurrence after treatment with HPIs may be difficult. As reported in a recent analysis, 31% of patients reporting a complete BCC clearance with 6 months of vismodegib showed tumor relapse after 6 months of therapy withdrawal.^5^ Recent evidences showed that recurrence after HPI discontinuation following response does not constitute resistance, therefore rechallenge with a drug of the same class may be considered.^6^, ^7^ Although a number of clinical studies highlighted the differences between the two approved HPIs, no head‐to‐head trial is available. However, sonidegib showed concentrations in the skin sixfold higher than in plasma and the approved alternate‐day dose regimen could increase patient's tolerability and compliance, thus leading to the extension of treatment duration and sustained benefit.^8^

## CONFLICT OF INTEREST

The authors declare no potential conflict of interest.

## Data Availability

Data sharing not applicable—no new data generated.
